# Solving the Problem: Genome Annotation Standards before the Data Deluge

**DOI:** 10.4056/sigs.2084864

**Published:** 2011-10-01

**Authors:** William Klimke, Claire O'Donovan, Owen White, J. Rodney Brister, Karen Clark, Boris Fedorov, Ilene Mizrachi, Kim D. Pruitt, Tatiana Tatusova

**Affiliations:** 1 The National Center for Biotechnology Information, National Library of Medicine, NIH, Building 45, Bethesda, MD 20894, USA; 2 UniProt, The EMBL Outstation, The European Bioinformatics Institute, Wellcome Trust Genome Campus, Hinxton, Cambridge CB10 1SD, UK; 3 Institute for Genome Sciences, University of Maryland School of Medicine, Baltimore, MD 21201, USA

## Abstract

The promise of genome sequencing was that the vast undiscovered country would be mapped out by comparison of the multitude of sequences available and would aid researchers in deciphering the role of each gene in every organism. Researchers recognize that there is a need for high quality data. However, different annotation procedures, numerous databases, and a diminishing percentage of experimentally determined gene functions have resulted in a spectrum of annotation quality. NCBI in collaboration with sequencing centers, archival databases, and researchers, has developed the first international annotation standards, a fundamental step in ensuring that high quality complete prokaryotic genomes are available as gold standard references. Highlights include the development of annotation assessment tools, community acceptance of protein naming standards, comparison of annotation resources to provide consistent annotation, and improved tracking of the evidence used to generate a particular annotation. The development of a set of minimal standards, including the requirement for annotated complete prokaryotic genomes to contain a full set of ribosomal RNAs, transfer RNAs, and proteins encoding core conserved functions, is an historic milestone. The use of these standards in existing genomes and future submissions will increase the quality of databases, enabling researchers to make accurate biological discoveries.

## Introduction

### Annotation Issues in Genome Records

Even before the first genome sequence for a cellular organism was completed in 1995, it was recognized that the functional content encoded by and annotated on nucleotide records represented both a blessing and a curse [1-3]. With the complete genome sequence obtained and annotated, a full understanding of the biology of an organism was thought to be within reach. However, deposition of an annotated record into the sequence archives, excepting the rare occasion when a record is updated, meant that the archival record represented a snapshot in time of both the sequence and annotation. Scientists have sought to address the annotation issue by creating curated databases, developing computational tools for the assessment of annotation, and publishing a variety of solutions in numerous papers [4,5].

Throughout the sequencing era, continuous reassessment of annotations based on new evidence led to improved annotations on a number of sequences, even though the process is recognized as being time-intensive [6,7]. With the exponential increase in sequence data, annotation updates have become increasingly unlikely events. Errors in annotation impact downstream analyses [8]. Errors that affect the location of annotated features or that result in a missed genomic feature greatly impact the evolutionary studies and biological understanding of an organism, whereas mistakes in functional annotation lead to subsequent problems in the analyses of pathways, systems, and metabolic processes. The presence of inaccurate annotation in biological databases introduces a hidden cost to researchers that is amplified by the amount of data being produced. For prokaryotic organisms, as of August 10, 2010, there were 1,218 complete and more than 1,400 draft genomes that had been sequenced and released publicly. The Genome Project database and other online efforts to catalog genome sequencing initiatives list thousands of additional sequence projects that have been initiated but for which sequence data has not yet been released [9,10]. Investigators relying on the complete genome set consisting of sequenced and closed replicon molecules and annotations as a gold standard are becoming increasingly affected by the size of the dataset even without having to take into account the presence of erroneous annotation [11]. As rapidly decreasing sequencing costs for next generation sequencing are producing unprecedented levels of data and errors that can easily inflate in size and propagate throughout many datasets, it is essential that steps be taken to address these issues [8,12].

A large body of literature devoted to describing annotation problems is available ([13,14] and references within). Errors that plague genome annotations range from simple spelling mistakes that may affect a few records, to incorrectly tuned parameters in automatic annotation pipelines that can affect thousands of genes. Discrepancies can impact the genomic coordinates of a feature, or the function ascribed to a feature such as the protein or gene name, or both [15]. The commonly used Gene Ontology annotations are also subject to errors [16]. As our understanding of genome biology and evolution has improved, a number of methods have been developed to assess annotation quality. Typically, several pieces of evidence are combined in order to assign confidence levels to a particular annotation or to predict new functions. In some cases these methods have led investigators to target a specific function for experimental validation after the prediction was made, a process that both validated the prediction method and provided improved and experimentally determined annotations such as in the detection of the GGDEF and EAL domains as a major part of prokaryotic regulation [17-19]. Some of these methods include sequence similarity, phylogenomic or genomic context, metabolic reconstruction to determine pathway holes, comparative genomics, and in many cases a combination of all of the above (reviewed in [20]). A number of tools have been developed to predict annotations based on curated and experimental data. Curated model organism databases or datasets for specific molecules such as transfer RNAs, ribosomal RNAs, or other non-coding RNAs have been developed along with tools to predict their presence in a novel sequence [21-24].

Several large-scale curated databases have been created at large centers, such as at EBI and NCBI. NCBI initiated the Reference Sequence database to create a curated non-redundant set of sequences derived from original submissions to INSDC [25]. The sequences include genomic DNA, transcripts, and proteins and the annotations may consist of submitter-derived, curated, or computational predictions. One major resource for improving functional annotation is the NCBI Protein Clusters database that consists of cliques of related proteins (ProtClustDB [26]; ). A subset of clusters are curated and utilized as sources of functional annotation in the annotation pipeline as well as to incrementally update RefSeq records (see below). RefSeq records are also updated from model organism databases such as those for *E. coli* K-12 or Flybase. The UniProt Knowledgebase (UniProtKB) provided by the UniProt consortium is an expertly curated database, a central access point for integrated protein information with cross-references to multiple sources [27]. The Genome Reviews portal that was a comprehensively up-to-date set of genomes has now been incorporated at ENSEMBL genomes [28,29]. Ongoing collaboration between NCBI and EBI ensures that annotation will continue to be curated and improved in all databases.

RefSeq is committed to ensuring that all current and future RefSeq prokaryotic records meet the minimal standards presented in this article. However, high throughput next generation sequencing increasingly results in a large number of non-reference sequences populating the databases with the expectation that there could be tens of thousands of genomes available for all prokaryotes. Community acceptance of a set of minimal annotation standards puts the burden on all genome submitters to provide quality annotation especially for those complete genomes that are often considered gold standard records for sequencing and annotation such as *Escherichia coli* K-12 MG1655.

### The Need for Standards

Standards and guidelines facilitate the submission, retrieval, exchange, and analysis of data. Both the format and content of data can be standardized (syntactic and semantic). Syntactic standardization is easier to implement and enforce. The format and representation of genomic records has long been established and is not discussed in this article. Semantic standardization is more difficult. Standardization of the genomic content and annotation will facilitate analyses at the functional and systems levels, in other words, the biology will be easier to understand and to put into an evolutionary context which will have a real impact on how researchers approach scientific studies.

An explosion of documents for minimal standards in a variety of genomics, bioinformatics, and transcriptomics studies has occurred. Examples include the MIAME standards established for microarray expression studies, and the MIGS standards that were created to establish minimal metadata associated with genome sequencing projects [30,31]. There is now the Minimum Information for Biological and Biomedical Investigations (MIBBI) project that aims to comprehensively organize and collate all of these projects and BioDBcore, a community initiative for specifications of biological databases [32,33]. Although the reason for standards is clear, the enforcement of standards is a complex issue that remains to be resolved [34]. Community standards that are adopted by the organizations producing, archiving, and distributing the data will facilitate the usage and enforcement of these standards. Recognizing these growing problems, the National Center for Biotechnology Information (NCBI) organized three Genome Annotation Workshops in 2006, 2007, and 2010. Participants included members of the International Nucleotide Sequence Database Collaboration (GenBank, the European Nucleotide Archive (ENA), and the DNA Data Bank of Japan (DDBJ), scientists from the European Bioinformatics Institute (EBI) including those from UniProt Consortium (PIR/EBI/SIB), and members of organizations not involved in archiving data such as those from the American Society for Microbiology (ASM), investigators from a variety of sequencing centers such as the Department of Energy's Joint Genome Institute, representatives associated with the NHGRI human microbiome project, and individual scientists. The first two workshops were aimed at resolving annotation problems for the growing numbers of prokaryotic genomes while the 2010 workshop brought together researchers from both the prokaryotic and viral fields. This report is a summary of the results from all three meetings. URLs for specific databases, tools, websites, guidelines, and documents can be found in Table 1 and the full set of links, updates, and contact information will be posted at the workshop site at NCBI [51].

**Table 1:** Databases, tools,resources for genomes and annotation.

**Category/Title**	**Description**	**Reference**	**URL**
**General**			
NCBI Genome Annotation Workshop	All information from this publication, the Annotation Workshop, and futureannouncements will be made available		http://www.ncbi.nlm.nih.gov/genomes/AnnotationWorkshop.html
Difference between Archive and Curated Databases	GenBank, RefSeq, TPA and UniProt:What’s in a Name?	Microbe Online	http://www.microbemagazine.org/index.php?option=com_content&view=article&id=1270:genbank-refseq-tpa-and-uniprot-whats-in-a-name&catid=347:letters&Itemid=646
Difference between Archive and Curated Databases	GenBank, RefSeq, TPA and UniProt:What’s in a Name?	NCBI Handbook	http://www.ncbi.nlm.nih.gov/bookshelf/br.fcgi?book=handbook&part=ch1#GenBank_ASM
INSDC	International Nucleotide Sequence Database Collaboration		http://www.insdc.org/
INSDC Feature Table	Feature table document		http://www.insdc.org/documents/feature_table.html
DDBJ	DNA Databank of Japan	[35]	http://www.ddbj.nig.ac.jp/
ENA	European Nucleotide Archive	[36]	http://www.ebi.ac.uk/ena/
GenBank	GenBank	[20]	http://www.ncbi.nlm.nih.gov/genbank/index.html
**Automated Annotation providers**			
NCBI Prokaryotic Genomes Automatic Annotation Pipeline (PGAAP)	Intended for use during the annotation of prokaryotic genomes in preparation for submission to GenBank – capable of annotating complete genomes as wells WGS genomes		http://www.ncbi.nlm.nih.gov/genomes/static/Pipeline.html
JCVI Annotation Service	Anyone with a prokaryotic genome sequence in need of annotation may submit it to the JCVI Annotation Service completely free-of-charge		http://www.jcvi.org/cms/research/projects/annotation-service/overview
IGS Annotation Engine	a free resource for genomics researchers and educators bringing advanced bioinformatics tools to the lab bench and the classroom.		http://ae.igs.umaryland.edu/cgi/index.cgi
KAAS - KEGG Automatic Annotation Server	KAAS (KEGG Automatic Annotation Server)provides functional annotation of genes by BLAST comparisons against the manually curated KEGG GENES database with resulting KO (KEGG Orthology) assignments and automatically generated KEGG pathways	[37]	http://www.genome.jp/tools/kaas
RAST	RAST (Rapid Annotation using Subsystem Technology) is a fully automated service for annotating bacterial and archaeal genomes –provides high quality genome annotations for these genomes across the whole phylogenetic tree	[38]	http://rast.nmpdr.org
DOE-JGI MAP	Expert Review Data Submission: Microbial Genomes & Management	[39]	http://img.jgi.doe.gov/cgi-bin/submit/main.cgi
**Annotation Cleanup, Analyses, and Validation Tools**			
NCBI Submission Check Tool	For the validation of genome submissions to GenBank –utilizes a series of self-consistency checks as well as comparison of submitted annotations to computed annotations - web-based and downloadable versions available		http://www.ncbi.nlm.nih.gov/genomes/frameshifts/frameshifts.cgi
NCBI Sequin Validation	Sequin is a standalone tool for submitting and updating sequences	[20]	http://www.ncbi.nlm.nih.gov/Sequin/QuickGuide/sequin.htm
NCBI TBL2ASN	Command-line tool for automation of sequence records to GenBank	[20]	http://www.ncbi.nlm.nih.gov/Genbank/tbl2asn2.html
NCBI Discrepancy report	Evaluation of ASN.1 files for annotation discrepancies- part of Sequin, available separately as downloadable command line version, and part of tbl2asn	[20]	http://www.ncbi.nlm.nih.gov/Genbank/asndisc.html
Broad's Gene Pidgin (formerly BioName)	Pidgin is a suite of tools that evaluate and automatically assign gene product names - standardization, comparison, and then selection of the best name		http://genepidgin.sourceforge.net/
JCVI's Protein Naming Utility	To address the need to generate high-quality protein names - a web-based database for storing and applying naming rules to identify and correct syntactically incorrect protein names, or to replace synonyms with their preferred name	[40]	http://www.jcvi.org/pn-utility/
Frameshift Tool	Frameshift detection in protein coding genes.	[41]	http://topaz.gatech.edu/GeneTack/
Annotation Report	Quantitative report on genome annotation		http://www.ncbi.nlm.nih.gov/genomes/annotreport.cgi
**Annotation Guidelines**			
GenBank Bacterial Genome Submission Guidelines	Bacterial genome specific submission guide	[20]	http://www.ncbi.nlm.nih.gov/Genbank/genomesubmit.html
Annotation Instructions	Detailed instructions on bacterial genome submissions	[20]	http://www.ncbi.nlm.nih.gov/Genbank/genomesubmit_annotation.html
Project Submission	Bioproject submission form	[42]	http://www.ncbi.nlm.nih.gov/genomes/mpfsubmission.cgi
Locus_tag proposal	Accepted locus_tag standards for INSDC		http://www.ncbi.nlm.nih.gov/genomes/locustag/Proposal.pdf
UniProt's Protein Naming Guidelines	UniProt's general protein naming guidelines		http://www.uniprot.org/docs/nameprot
UniProt's Protein Naming Guidelines - Prokaryotes	UniProt's prokaryotic-specific protein naming guidelines - adopted by INSDC		http://www.uniprot.org/docs/proknameprot
GSC Structured Format	Accepted structured format for genome metadata including SOPs	[43]	http://gensc.org/gc_wiki/index.php/MIGS/MIMS/MIENS
Insertion Sequences	Insertion sequence finder, nomenclature, and registry	[44]	http://www-is.biotoul.fr/
Transposons	Transposon nomenclature and registry	[45]	http://www.ucl.ac.uk/eastman/tn/
Enzyme Commission Numbers	Official NC-IUBMB site		http://www.chem.qmul.ac.uk/iubmb/enzyme/
UniProt ENZYME	ENZYME is a repository of information relative to the nomenclature of enzymes.		http://ca.expasy.org/enzyme/
**Functional Annotation/Protein Families**			
NCBI COGs	Clusters of orthologous groups - no longer actively curated	[46]	http://www.ncbi.nlm.nih.gov/COG/
NCBI ProtClustDB	Cliques of related proteins - curated and uncurated –for multiple organism groups including prokaryotes and Viruses	[33]	http://www.ncbi.nlm.nih.gov/proteinclusters
NCBI Cluster Comparison Tool	Protein family comparison for functional annotation		http://www.ncbi.nlm.nih.gov/sutils/clustcomp.cgi
NCBI Cluster Comparison Tool - Core Mode	Protein family core comparison for functional annotation		http://www.ncbi.nlm.nih.gov/sutils/clustcomp.cgi?core=on
List of Core Clusters	Protein family core list		http://www.ncbi.nlm.nih.gov/sutils/clustcomp.cgi?report=core
UniProt HAMAP	system, based on manual protein annotation, that identifies and semi-automatically annotates proteins that are part of well-conserved families or subfamilies in prokaryotes and plastids	[47]	http://ca.expasy.org/sprot/hamap/
KEGG Orthology Groups	Manually defined ortholog groups that correspond to KEGG pathway nodes and BRITE hierarchy nodes	[48]	http://www.genome.jp/kegg/ko.html
JCVI's TIGRFAMs	Protein families based on Hidden Markov Models	[49]	http://www.jcvi.org/cms/research/projects/tigrfams/overview/
ACLAME	Database dedicated to the collection and classification of mobile genetic elements	[50]	http://aclame.ulb.ac.be/
E. coli CCDS Project	Comparison of annotation for model E. coli K-12 MG1655		http://www.ncbi.nlm.nih.gov/genomes/MICROBES/ecok12.cgi

Milestones from all three workshops include: 1) the *E. coli* CCDS project (ECCDS), 2) a publication detailing the differences between archival and curated databases, 3) a locus_tag registry, and 4) release of a set of annotation assessment tools. Specific proposals on problems of genome annotation were generated from a number of working groups and focused on the following issues: 1) standard operating procedures, 2) structured evidence, 3) structural annotation, 4) pseudogenes, 5) protein naming guidelines, 6) comparison of functional annotation, 7) and viral annotation. Several of these proposals were submitted as guidelines and standards to be approved by INSDC while others are already accepted. Some of the proposals include reports and data sources that are available online (Table 1). The outcomes of each are summarized below.

### ECCDS

The human genome CCDS project, an active collaboration project between EBI, NCBI, Sanger, and UCSC, was established to create a core set of consistently annotated protein coding genes [52]. This project has now grown to include the mouse genome, and there are considerations for expanding this to other eukaryotic organisms. Using this project as a model, the *E. coli* consensus CDS project was established to reconcile the annotation differences for the model organism *E. coli* K-12 MG1655 which was first sequenced in 1997 (GenBank Accession Number U00096 [53]; ). An updated annotation snapshot was released in 2006, and numerous curated and archival databases contain annotation for this organism [43]. Of those, the ones actively contributing to the ECCDS project include GenBank, RefSeq, EcoGene, EcoCyc, and UniProt [25], [27] [54-56]. Consistent annotation has been established between EcoGene, GenBank, and RefSeq with all three synchronizing the annotation several times a year. Reconciliation of this consistent annotation set with the EcoCyc and UniProtKB/Swiss-Prot databases is an ongoing process that has resulted in improved annotations in all five databases benefiting not only *E. coli* researchers but also the entire field of prokaryotic genomics (Table 1).

### Differences between Archival and Curated Databases

Archival and curated databases serve different needs for the genomic and bioinformatics communities, but there is still confusion about the exact roles of all of these databases in the representation of genome sequencing data. A short article (“GenBank, RefSeq, TPA and UniProt: What’s in a Name?”) clarifying these issues was authored by NCBI and published in the ASM journal Microbe and is also available online at NCBI (Table 1). The article discussed the differences between the archival databases (GenBank), curated databases such as RefSeq and UniProtKB/Swiss-Prot, and Third Party Annotation (TPA), and helped researchers to understand the exact role of each database and how sequences and annotations are handled in each. Archival databases such as GenBank contain primary submissions and redundant sequences whereas the TPA database provides the ability for peer reviewed and published information to be used to update the information in the primary archives. RefSeq and UniProt have been described above. These resources constitute a major part of the dataflow for the annotation, submission, retrieval, and analysis of genomic records.

### Locus_tag registry

Locus_tags are systematic identifiers used for the enumeration of annotated genes even for cases when the genes have no known function. ASM journal editors had noticed that there was an increased use of locus_tags to refer to genes in the scientific literature, both in the primary genome sequencing paper as well as in subsequent publications describing specific genes and functions. However, as these identifiers were annotated by individual investigators and research labs, there were increasing instances of the same locus_tag being used to describe different but unrelated genes in different organisms. Hence the utility of a unique identifier was being lost and the use of locus_tags in a scientific article to identify particular genes was resulting in confusion. The solution was to create a locus_tag registry in conjunction with the Genome Project (soon to be BioProject [57]) database. Prefixes consisting of alphanumeric characters that met the standards could be registered along with a genome project submission (Table 1). The assignment of a unique locus_tag prefix to each genome assures that each gene feature in the dataset of all genomes records can be correctly identified.

### Annotation Assessment Tools

NCBI committed to produce additional annotation assessment tools to help submitters find problems with genome annotations (Table 1). These tools are used during the submission process to GenBank, in the Prokaryotic Genome Automatic Annotation Pipeline, and are available separately and include: 1) the Discrepancy Report which includes internal consistency checks without the use of external databases, and is available in Sequin, as part of the tbl2asn tool or as a stand-alone command-line tool, 2) the subcheck/frameshift tool which incorporates sequence searches in external databases during annotation assessment in order to find potentially frameshifted genes and other annotation issues and is available via the web or as a command line tool. NCBI encourages submitters to utilize these tools prior to submission to aid in the identification and correction of annotation discrepancies. A new annotation report that lists quantitative annotation measures and provides comparison with multiple organisms is also available and is detailed below.

### Capturing Annotation Methods and Information Sources

The results of genome annotation processes are deposited along with sequence records in the archival databases. The combination of methods and information sources that were used in the creation of a particular genome annotation are usually detailed in a publication. With increasing numbers of genomes being deposited that do not have an associated scientific publication, it is of paramount importance that there is a process to capture the methods and databases used in creating a set of annotated features.

### Standard Operating Procedures

Standard Operating Procedures (SOPs) in the context of genome annotation should: 1) document specific processes used to generate annotations, 2) with enough detail to replicate the process, 3) list the input and outputs, 4) reference any external tools, and 5) and describe how the outputs of software packages are interpreted, filtered, or combined. The concept of SOPs, along with an example using the NCBI prokaryotic genome automatic annotation pipeline (PGAAP), has been detailed elsewhere [58]. The Genome Standards Consortium (GSC), which has set forth a structured format to capture genome metadata, provides optional fields to link to an online accessible SOP via a digital object identifier (DOI) or other mechanism [31]. INSDC has agreed to adopt this structured format for genome metadata, thus providing the capability to document SOPs and link them to each genome record with the metadata appearing in the COMMENT section. An example record with structured metadata can be found in GenBank Accession Number CP002903 (although the annotation SOP is not yet provided for this particular genome). All submitters are encouraged to use this structured format to capture genome metadata.

### Structured standards evidence in annotation

SOPs describe the processes used to make an annotation decision including a list of information sources which may include sequence, structure, domain databases, or protein family resources. Since many of these bioinformatics sources are large databases with many records, it is essential to note the exact record from which an annotation is derived, thus providing a one-to-one or many-to-one link from annotation sources to the novel predicted annotation in a new genome. The source becomes a vital reference that facilitates analysis and comparison and the link to a particular record provides a trail through which annotation updates or problems can be addressed.

A variety of evidence or confidence-based systems are currently used. The Evidence Viewer at NCBI displays the sequences that provide evidence for the sequence of a particular gene model or mRNA [42]. The RefSeq status key provides varying levels of confidence to a particular annotation based on the level of manual review a particular annotation has received [25]. The curated *Pseudomonas aeruginosa* database incorporates evidence levels for functional assignments [59]. UniProt has developed an evidence attribution system which attaches an evidence tag to each data item in a UniProtKB entry identifying its source(s) and/or methods used to generate it. Users can easily identify information added during the manual curation process, imported from other databases or added by automatic annotation procedures. In addition, UniProt has developed the protein existence concept which provides the level of evidence available for the existence of a protein [27]. The Gene Ontology (GO) system provides evidence for function, component, and process and is one of the better known systems used in annotation today [60]. However, GO cannot be used for all features on a genome, nor are all genome sequencing centers and large-scale institutes routinely using GO or any of the other ontologies, and similar issues arise with all of the above-mentioned evidence systems.

The INSDC flatfile is a commonly used format. It provides the capability to annotate many features such as genes, protein-binding sites, or ribosomal RNAs. For each feature there is a set of mandatory and optional qualifiers (Table 1) that provide detailed information in a structured format for each particular feature. For example, the gene name, the protein binding the DNA, or the ribosomal RNA product. The flatfile format is reviewed every year by the member databases and proposed changes are discussed before acceptance.

The evidence used to annotate a particular feature can be encapsulated in two optional qualifiers, “/experiment” and “/inference”. Whereas the “/experiment” qualifier provides information on the nature of the experiment used to derive the annotation of a particular feature, for example N-terminal sequencing to determine the peptide sequence, the “/inference” qualifier provides information on the non-experimental evidence to support the annotation of a particular feature. Three tokens have been proposed and accepted that further categorize the two annotation qualifiers: 1) existence, 2) coordinates, 3) description, and additionally the experiment qualifier provides a field for a direct link to a PubMed identifier or DOI detailing the experiment where support for one of the three tokens can be found (Table 2). A combination of the three tokens can be applied to a set of qualifiers on a feature. For example, the evidence for the exact start and stop of a protein coding region for a particular organism is experimentally determined in one publication while the function is derived by inference from a related organism and all of the evidence and the sources used to derive each annotation can be captured with the set of qualifiers and tokens.

**Table 2:** Summary of structured evidence for INSDC feature annotation^1^

	Allowed tokens		
**Token**	**"/experiment" qualifier^2^**	**"/inference" qualifier^3^**	**Comment**
free text	Yes	No	free text describing the experiment
non experimental structured format	No	Yes	structured format of TYPE + EVIDENCE_BASIS (type includes “non experimental”, “similar to”, “profile”, or “alignment”, evidence basis can include algorithm with version, or database with accession.version)
**coordinates**	Yes	Yes	support for annotated coordinates
**description**	Yes	Yes	support for description including function
**existence**	Yes	Yes	support for existence of feature in this organism
**PMID or DOI**	Yes	No	publication describing experimental evidence

1. Changes proposed and accepted by INSDC to the /experiment and /inference qualifiers. The new tokens (bolded) are optional for both qualifiers.

2. A brief description of the nature of the experimental evidence that supports the feature identification or assignment.

3. A structured description of non-experimental evidence inferring and supporting feature identification or assignment.

This system of evidence linkage gives richer context to genome annotation where the evidence and processes used to derive annotation is completely traceable. RefSeq will begin implementing evidence assignments and encourages all genome researchers to do the same. Mechanisms for the search, retrieval, and subcategorization of genome records and features with different levels of evidence will be provided by the major databases.

## Structural Annotation

### Structural annotation and gene calling standards, validation (reports and outcomes)

**Figure 1:**
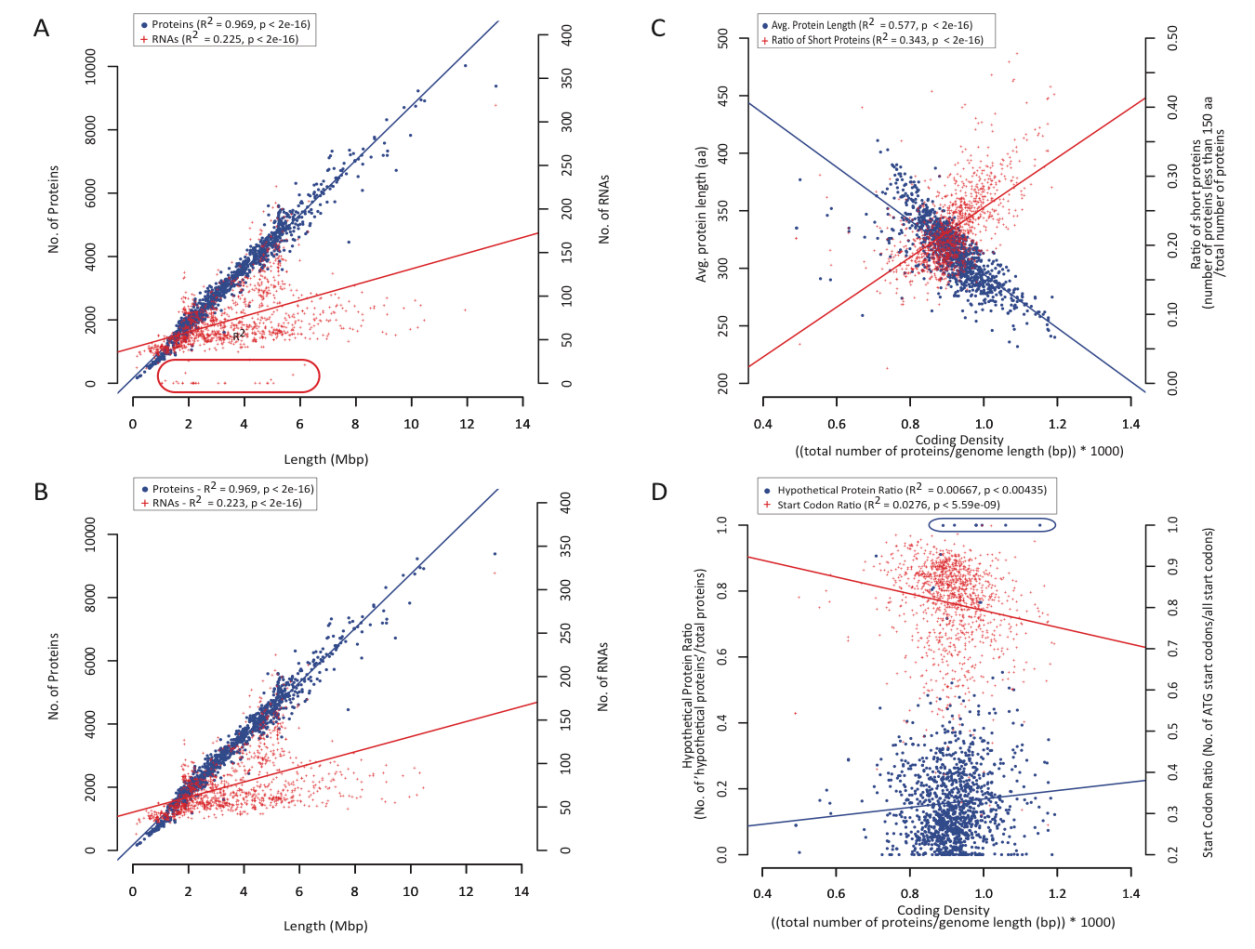
Selected comparisons of genome measures. Principal component analysis showed expected relationships among the different measures (data not shown). Selected examples are plotted as double y-axis scatterplots. Legends indicate first or second y-axis for blue dots or red crosses, respectively. Linear regression analysis of each y-axes variable independently with respect to the x-axis variable was done and the trend line is drawn on each plot color-coded with respect to each measure. R^2^ and p-values are shown for each measure. A-B. Numbers of annotated proteins and RNAs with respect to genome size from INSDC and RefSeq annotation sets for complete prokaryotic genomes. Feature counts were obtained from the Complete Microbial Genomes Annotation Report (Aug 10, 2010) and proteins and RNAs from INSDC and RefSeq are plotted with respect to genome length. The count of proteins follows a linear increase with respect to increasing genome size (blue trend line) while the RNA count, which includes all transfer, ribosomal, and non-coding RNAs, shows less of an increase with respect to genome size. Some genomes have extensively annotated RNA features, whereas others do not. A. All INSDC genomes (total of 1218 as of Aug 10, 2010). Those records that have below minimal standards for essential RNAs are encircled (red ellipse). B. RefSeq genomes (total of 1148 genomes as of Aug 10, 2010). Note, not all INSDC genomes are copied into RefSeq records. For the cases where INSDC records were missing essential RNAs, if there was a RefSeq version, the essential RNAs have been added or properly labeled. In all cases where the full set of essential RNAs could not be annotated it appeared that the missing RNA(s) were either non-functional or completely missing from the genome sequence (Table 3; data not shown). C. Protein lengths with respect to coding density for INSDC annotations. As coding density increases (more proteins per Kbp) the average protein length decreases (blue trend line) and the ratio of short proteins increases (red trend line). D. Hypothetical proteins and start codon ratios versus coding density. The ratio of proteins named 'hypothetical' increases slightly as the coding density increases whereas the standard start codon ratio decreases. Genomes where 'hypothetical protein' ratio is 1 or near 1 (large blue ellipse - every protein is annotated as 'hypothetical protein' in the genome) falls below the minimal annotation standards. For these particular cases, if a RefSeq version of the annotation existed, the functional assignment of a number of proteins was improved via curated clusters in the NCBI ProtClustDB (data not shown).

Structural annotation standards refer to the methods and parameters used to call and validate genes on a genome. Numerous research laboratories and sequencing centers utilize a variety of different annotation methods and sources and those should be captured as noted above. Therefore, a specific set of software tools or databases was not chosen as a gold standard set. Instead, a non-exhaustive set of software tools and resources that produces high quality annotations and that are publicly available are listed (Table 1) and will be available online [51]. Researchers interested in annotating genomes are encouraged to start with this list. Quantitative measures of annotation were implemented to institute a set of minimal standards. Irrespective of the methodology and datasets used to annotate a particular genome, there are certain aspects of genome biology that are expected to be present for all prokaryotes. Key functions that should be present in all genomes include a set of core genes/functions as well as a complete set of ribosomal RNAs and transfer RNAs that are required for protein translation [61,62]. These requirements are detailed in the minimal standards below and are expected to be found on all complete genomes. Simple statistical reporting of various genome annotation measures can also be used to assess annotation quality. For example, the distributions of protein lengths reflects evolutionary constraints and an examination of length versus conservation showed that conserved genes tend to be longer than non-conserved [63]. Except for extreme cases, most prokaryotic genomes should exhibit similar genome characteristics and be within an expected distribution for each measure. Evolutionary forces that may drive a particular genome outside of an expected range of values include processes such as genome degradation in obligate intracellular endosymbionts or decreasing intergenic spacer size due to genome streamlining in ubiquitous ocean microbes [64,65]. NCBI now generates reports that allows comparison against publicly available genomes and will provide a similar report to all genome submitters in an effort to identify and correct annotation problems before a genome is publicly released (Table 1). Examples of these statistics are shown in Table 3. Two model organisms, *E. coli* and *Bacillus subtilis*, were chosen to represent well-annotated average genomes. All other genomes in the table exhibit extremes (minimum or maximum) for a particular category, and in some instances this reflects annotation that does not meet the minimum standards. In cases where a RefSeq copy of a genome was made, corrected annotations were added so that the minimum requirements were met. Comparison of selected annotation measures for all organisms is shown in Figure 1. A selected set was used in principal component analysis to find those measures that contribute the most to variation, and to find clusters of annotation measures. The two physical measures are the length of the chromosomes and the GC content. All other measures are annotation-derived. Length affects all annotation metrics and is one of the main drivers of annotation variance. For example, an assessment of protein and RNA count for all genomes shows a linear increase of the number of proteins as the genome size grows (Figure 1). Non-coding RNAs (ribosomal, transfer, and non-coding RNAs such as antisense RNAs), exhibit less of a slope, and in several genomes in the INSDC archives no RNAs have been annotated at all (Figure 1A). In the complement of complete RefSeq genomes, the full set of ribosomal and tRNAs have been added either as functional or as potential pseudogenes (Figure 1B). The only cases where this minimal standard could not be met were due either to issues with the sequence (sequencing or assembly) or cases of real biology such as in small compact genomes for endosymbionts. For example, *Candidatus* Hodgkinia cicadicola Dsem is missing several key functional tRNAs due to codon recoding [66].

**Table 3:** Selected annotation report examples^1^

		**Chromosome**				**Feature counts**				**Calculated values**					
**Bioproject ID^2^**	**Organism Name**	**No. of replicons**	**Length (Mbp)**	**GC (%)**		**No. of proteins**	**No. of RNAs**	**No. of amino acids with tRNA^5^**		**No. of hypothetical proteins^3^**	**Coding Density^4^**	**Avg. protein length (aa)**	**Min. protein length (aa)**	**Short proteins [%]^6^**	**Percent standard start codon [%]^7^**
225	*Escherichia coli* str. K-12 substr. MG1655	1	4.640	50.79		4144	175	**22**		21	0.89	316	14	20.32	90.54
76	*Bacillus subtilis* subsp. subtilis str. 168	1	4.216	43.51		4177	178	20		221	0.99	294	20	26.48	77.76
17977	Candidatus *Carsonella ruddii* PV	1	0.160	**16.56**		182	31	20		44	1.14	274	37	32.42	96.15
32135	Candidatus *Hodgkinia cicadicola* Dsem	1	**0.144**	58.39		**169**	18	12*		37	1.18	257	38	33.73	**27.22**
46847	*Streptomyces bingchenggensis* BCW-1	1	11.937	70.75		**10022**	84	21		3606	0.84	342	24	19.86	60.69
19943	*Rickettsia rickettsii* str. Iowa	1	1.268	32.45		1384	37	19*		607	1.09	**232**	17	**47.76**	73.55
81	*Clostridium tetani* E88	1	2.799	28.75		2373	72	20		247	0.85	336	**101**	12.09	68.27
12634	*Anaeromyxobacter dehalogenans* 2CP-C	1	5.013	**74.91**		4346	58	21		965	0.87	349	38	15.85	69.21
49535	*Propionibacterium freudenreichii* subsp. shermanii CIRM-BIA1	1	2.616	67.27		2375	51	20		721	0.91	317	**2**	21.14	70.57
43535	*Lactobacillus salivarius* CECT 5713	1	1.828	32.94		1350	120	21		86	0.74	352	95	**2.22**	80.00
105	*Haloarcula marismortui* ATCC 43049	2	3.420	61.93		3412	59	20		1	1.00	285	30	27.02	**100.00**
13128	*Photobacterium profundum* SS9	2	6.323	41.71		5413	209	21		2490	0.86	316	35	21.97	73.88
28711	*Haliangium ochraceum* DSM 14365	1	9.446	69.48		6719	55	20		1827	0.71	**411**	32	13.37	79.67
244	*Nostoc* sp. PCC 7120	1	6.414	41.35		5368	64	20		**0**	0.84	326	17	25.58	82.41
19857	*Vibrio harveyi* ATCC BAA-1116	2	5.969	45.44		5944	159	20		5944*	1.00	286	24	30.43	84.84
28111	*Sorangium cellulosum* 'So ce 56'	1	**13.034**	71.38		9375	**319**	0*		4170	0.72	401	30	13.08	73.33
344	*Rhizobium leguminosarum* bv. viciae 3841	1	5.057	61.09		4700	0*	0*		247	0.93	309	40	19.57	80.83
31271	*Mycobacterium leprae* Br4923	1	3.268	57.80		1604	47	20		143	**0.49**	335	33	21.01	54.30
29335	*Neisseria gonorrhoeae* NCCP11945	1	2.232	52.37		2662	67	20		324	**1.19**	240	32	41.81	71.22

1. Selected genomes and categories for INSDC genomes are shown. The first two rows are for the model organisms E. coli and B. subtilis. The other genomes were selected as the minimum (bolded) or maximum (bolded and underlined) in the categories shown. Those marked with an asterisk fall below the minimal standards described in this publication.

2. INSDC Bioproject ID for each genome [57].

3. Number of proteins annotated as 'hypothetical protein'.

4. Number of proteins per Kbp ((total number of proteins/genome length (bp)) * 1000).

5. Number of amino acids for which at least one tRNA is annotated in the genome (excluding predicted or annotated pseudo tRNAs).

6. Percent of short proteins (number less than 150 amino acids in length/total number of proteins * 100).

7. Percent of standard starts for proteins (number of standard starts (ATG)/total starts * 100).

**Figure 2:**
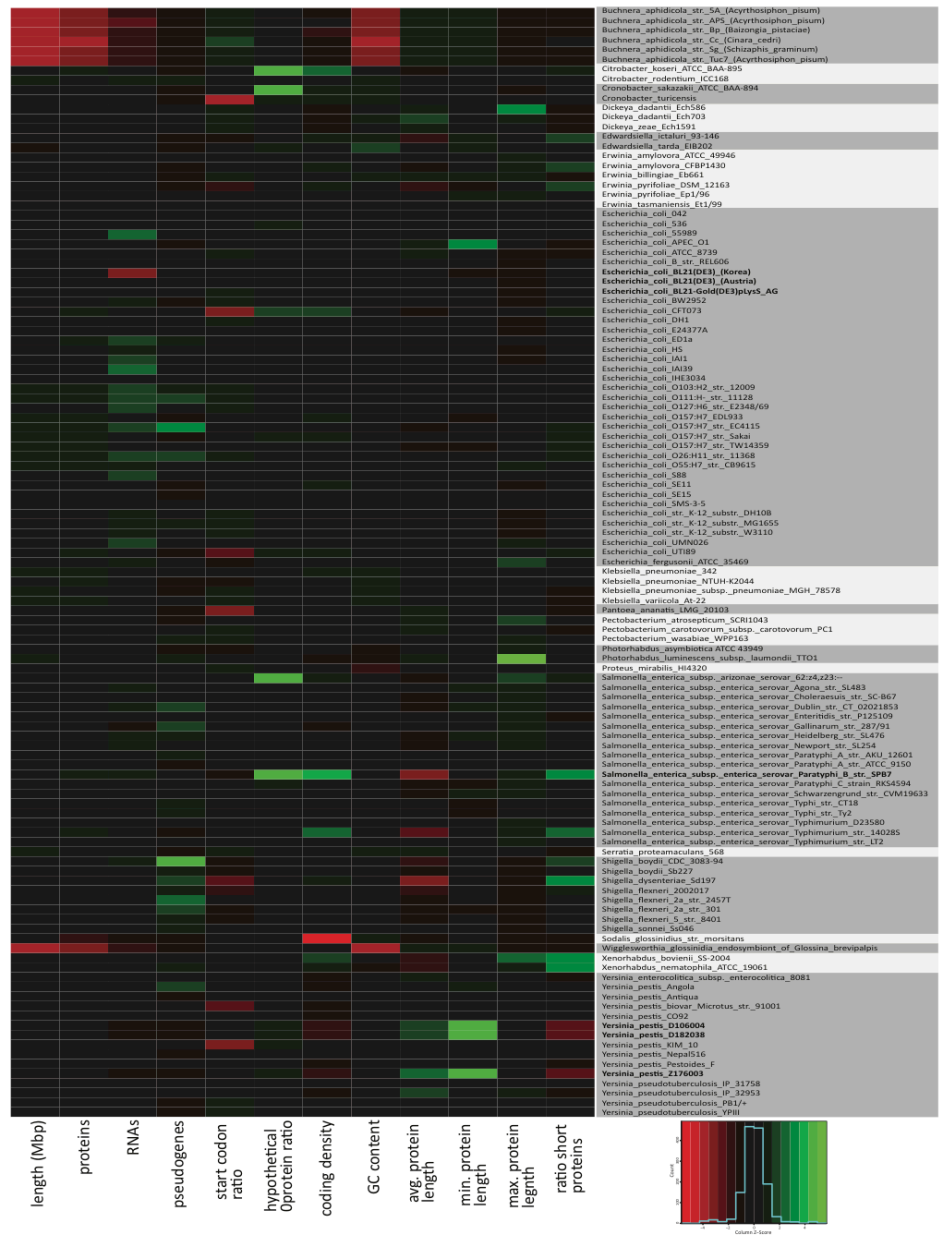
Heatmap of selected annotation report measures for gammaproteobacteria. A set of measures were chosen corresponding to those used in principal component analysis (data not shown) but restricted to INSDC genomes from gammaproteobacteria. A two-dimensional clustering of the selected and scaled data (subtracted column means, division by standard deviation) demonstrates similar clusters that were obtained in the PCA analysis (data not shown). For Figure 2, no clustering was done and the input genomes are arranged alphabetically by organism name and shaded to indicate different genera. A color-key and histogram at bottom right indicate the relative intensities of the annotation measures (the histogram applies to all measures, color intensities apply to each cell). Genomes described in the text are in bold.

Further examination of the annotation measures across all genomes shows how other measures interact. For example, increasing coding density (more genes per Kbp) in genomes results from an increase in the ratio of short proteins (ratio of proteins that are less than 150 amino acids/ total proteins: Figure 2C). As the coding density increases and the ratio of short proteins increase, the average protein length decreases, a logical result as the increased coding density is due to an increase in short overlapping predicted ORFs. A more subtle impact shows that with increasing coding density the ratio of hypothetical to total proteins in the genome increases, whereas the utilization of the ATG start codon (standard start) decreases (Figure 2D). Increasing GC content also coincides with the usage of alternative start codons such as GTG. However, increasing GC content and increasing genome length do not generally result in an increase in the hypothetical protein ratio (data not shown) suggesting that these trends are due to differences in annotation quality.

Although genome streamlining can impact these measures, for example many genomes from the *Prochlorococcus* genus exhibit increased coding density; there are other factors at play [64,67,68]. This is more clearly seen when closely related genomes are compared as in a heatmap [69]. Selected annotation measures for the gammaproteobacteria are compared in a heatmap in Figure 2. In several cases, increases or decreases in physical (length, GC content) or derived measures are due to biological causes. For example, gammaproteobacterial endosymbionts such as *Buchnera* spp. exhibit reduced genome size and decreased GC content [70,71]. In other cases a particular strain or set of strains exhibit skewed annotation measures as compared to other genomes of the same species. For example, one particular *Salmonella* genome exhibits an increased coding density, ratio of short proteins, and number of hypothetical proteins along with a decreased average protein length (*Salmonella enterica* subsp. *enterica* serovar Paratyphi B str. SPB7). In other cases subclusters of a particular species are formed due to potential erroneous annotations such as the three *Yersinia pestis* genomes that cluster separately from other *Y. pestis* strains due to skews in annotation that were derived from the same pipeline [72]. In other cases, substrains do not cluster together as the annotations were derived from three different annotation pipelines such as the case for *E. coli* BL21 where three isolates were sequenced and annotated by three different research groups [73]. Evolutionary events that result in altered annotations in a particular organism are significant and aid our understanding of the biology of not only that particular organism but of related organisms. Annotation differences due to the utilization of different methods and sources skew these results and the conclusions that result from them.

Researchers are encouraged to update their annotations on archival records to meet the minimal standards and to correct any annotation discrepancies. Systems are being developed at NCBI to check newly submitted genomes for compliance with minimal standards and reports will be provided to submitters for quality assurance. Genomic records where the minimal standards cannot be met for real biological reasons will have explanatory comments added to the record.

### Pseudogene Identification, Nomenclature, and Annotation

Pseudogene definitions take a variety of forms and the difficulties in properly defining and labeling pseudogenes stem from the same problem: **a negative cannot be experimentally verified [**74**]**. In eukaryotes, pseudogenes are defined as non-functional copies of gene fragments due to retrotransposition or genomic duplication, while in prokaryotes they result from degradation processes of either single copy or multiple copy genes either after duplication or failed horizontal transfer events [74,75]. A recent analysis of pseudogenes in *Salmonella* genomes suggests that they are cleared relatively rapidly from a genome indicating that their presence is a recent evolutionary event [76]. Although a clear definition of pseudogenes was not put forth, it was stressed that INSDC expects that all genome annotation should reflect the biology as determined by the underlying sequence. The INSDC feature table format provides several exceptions for cases of unusual biology but there are consequences for these unusual annotations that serve as flags in genome records (Table 3). A proposal was made to alter the pseudogene qualifier "/pseudo" to both"/pseudogene" and "/nonfunctional" as /pseudo is not considered to equate 100% to /pseudogene and that request is still being discussed by INSDC. The INSDC submission guidelines as they currently stand and the possible annotation strategies for pseudogenes, non-functional genes, and other cases are detailed in Table 4. It is essential for the research community to understand that in all cases, **INSDC does not allow a translated product (protein or polypeptide chain) to be derived from a feature labeled as a pseudogene.** More specifically, an instantiated peptide sequence, a product, and protein identifiers are not allowed for annotation purposes. Similarly, gene fragments (regions of similarity without valid start and stop) may not be annotated with translations. Exceptions to these rules require specific qualifiers that must fit specified formats and requirements.

**Table 4:** Pseudogene annotation strategies and outcomes

**Case**		**Situation**	**Flag^1^**	**How to Annotate**	**Consequence^2^**	**Protein Sequence Encoded/ Present in BLAST^3^**
1		Pseudogene	"/pseudo"	pseudogene	no translation; product name is in note, associated feature (CDS, tRNA, rRNA, etc.) will be annotated	No
2		Potential pseudogene	N/A	normal gene annotated, potential pseudogene status in note	no CDS feature, not documented as a pseudogene, not trackable as protein vs. RNA-coding	No
3a		Frameshifted gene and sequence **IS** correct	"/pseudo"	combine intervals into a single gene with /pseudo	no translation; product name is in note	No
3b		Frameshifted gene and sequence **MAY** be correct	N/A	keep both and add a note to each CDS	two separate coding regions and two protein translations	Yes (Both)
3c*		Frameshifted gene and there are sequence **ERRORS**	/”exception=”annotated by transcript or proteomic data” AND ("/experiment" OR "/inference")	experimental evidence defining the evidence that translation is correct and/or inference pointing to Accession Number with correct translation	protein sequence imported- translation does not match nucleotide	Yes
3d		Frameshifted gene and there are sequence **ERRORS**	"/artificial_location"	locations altered for 'correct' location	all protein deflines prefaced with “LOW-QUALITY PROTEIN:”	Yes
4		Region of similarity	N/A	misc_feature denoting location of region of similarity	no gene, no locus_tag, not systematically enumerated	No
5		Potential unresolvable problems	N/A	note explaining the issue	no change in annotation	Yes
6^4^		Split/interrupted gene in the case of an insertion (ex. transposon insertion)	N/A	could be either a single interval, or a split interval, annotation depends on consequence of insertion	no standards for split genes, locations do not match regions of similarity	No

1. Qualifier to be used on feature.

2. Downstream consequence of annotation decision, including impacts on presentation of the record.

3. Whether a protein sequence is encoded and will be present in protein and BLAST databases. Note, BLAST dbs only provide the ability to differentiate proteins based on defline changes. ie. Case 3b, 3c, and 5 present undifferentiated protein deflines in BLAST databases whereas case 3d has an altered protein defline.

4. Insertions can result in complicated cases such as gene fusion events. These annotation results should be due to real insertions, not simply regions of the genome that exhibit weak similarity to a part of a protein sequence.

## Functional Annotation

Functional annotation results include guidelines on protein naming as well as a project to compare different protein naming resources in an effort to converge towards a consistent set of protein names by utilizing common guidelines.

### Functional Annotation - Protein Naming Guidelines

Establishing protein naming standards has been a keystone of various curation efforts. In particular, this issue recognizes the protein name as the lowest common denominator of information exchange. The protein name is what is used in BLAST definition lines, which many users utilize as the sole information source. Ontologies were discussed but were not considered a priority. Ensuring up-to-date and well formatted protein names aids functional comparison and reliable hypotheses can be generated based on a set of consistent names, while the converse is true for badly formed names. UniProt had established publicly available naming guidelines that were modified during discussions and a set of prokaryotic-specific naming guidelines was adopted. The guidelines provide a basis for efficient and effective protein naming that is being used in the curation of both UniProt and RefSeq annotations. It is expected that all genomes submitted to INSDC will also follow these guidelines. A separate publication will detail the UniProt naming guidelines which are currently available online (Table 1). In addition, there is a general functional naming guideline that is applicable to protein names for all organisms (Table 1).

One particular issue of protein naming is the issue of specific names for proteins that have unknown or uncertain functional assignments. The final accepted resolution is that only two synonymous names will be acceptable: “hypothetical protein” or “uncharacterized protein”. Names such as “conserved hypothetical protein”, “novel protein”, or “protein of unknown function” are no longer acceptable in genome submissions.

### Comparison of functional annotation sources

Numerous resources are used in the annotation of protein functions and names and there are two established models for curation. Either a model organism database has been established for particularly important or well-studied organisms, or a set of protein families with similar function have been curated. One of the earliest examples of the latter was the Clusters of Orthologous Groups developed at NCBI which is no longer actively curated [46]. Since that time extensive work has been done by at least four separate groups: JCVI has produced the TIGRFAM set of protein families with a subset identified as equivalogs with the same function, UniProt's High-quality Automated and Manual Annotation of microbial and chloroplast Proteomes (HAMAP), the Kyoto Encyclopedia of Genes and Genomes (KEGG) orthology groups (KO) that uses NCBI Reference Sequences, and NCBI's Protein Clusters database that includes prokaryote, viral, and selected eukaryotic organism groups (ProtClustDB) [26], [46,47,49,77]. The TIGRFAMs and HAMAP projects contain only curated families, whereas KEGG and ProtClustDB have both curated and uncurated clusters. In 2009 NCBI and JCVI jointly collaborated on an initiative to compare the functional names derived from TIGRFAMs with NCBI's curated protein clusters. The comparison results led to improvements in both databases (data not shown). A comparison of protein family annotation from all four databases is available online (Table 1).

An immediate goal of this process was the establishment of a core functional set that is expected to be encoded in all genomes. A number of studies over the years have addressed the idea of a minimal set of essential functions for a prokaryotic organism. The exact number fluctuates depending on the set of organisms used, the criteria for determining orthology, and whether only complete proteins or domains are considered [61,62], [78]. The initial set of universal COGs derived from proteins encoded in the 66 unicellular genomes at that time served as a starting point. Correspondence to the NCBI protein clusters database was checked, and a preliminary set of 61 functions corresponding to 191 clusters was created [26,46]. Next, all complete RefSeq genomes were checked to determine if all core functions were encoded. For those genomes where a protein could not be found, the nucleotide sequence and annotation were examined to assess whether a pseudogene/frameshifted gene was already annotated that corresponded to the missed function. For those cases that did not already have an annotated feature, a proper translation of the missed gene was examined with the result that a number of core functions that were previously missed from the submitted genome annotation were added to the Reference Sequence record. A total of 42 protein coding genes and translated features were added covering 12 functional groups (Table 5). To determine if the proteins were missed due to their smaller size, an examination of their average length for the proteins found in clusters corresponding to these 12 core functions was undertaken. Although most of the core cluster sets exhibit average lengths that are less than the minimum of the range of average protein lengths found in all genomes (232 aa from Table 3), especially those that were most frequently missed such as ribosomal protein S14, most are above typical length cutoffs and should still be found in even the most rudimentary annotation pipelines. Therefore, high protein length thresholds during annotation pipeline runs cannot adequately explain all discrepancies and missed core functions. To help solve these problems, all new RefSeq genomes will be tested against the core set for missed functions, and this process will be made available both as a set of clusters and incorporated into existing genome analysis tools for submitters (Table 1). The core set will gradually be expanded to archaeal, bacterial, and then to more taxonomically restricted core functional sets such as species level pangenomic families [79].

**Table 5:** Core proteins added to RefSeq genomes^1^

**Protein^2^**	**Number of additions^3^**	**Avg. Length^4^**
30S ribosomal protein S8	1	131.4+/-2.1
30S ribosomal protein S11	1	130.1+/-5.8
30S ribosomal protein S14	10	84.1+/-19.3
30S ribosomal protein S15	3	94.1+/-17.1
30S ribosomal protein S19	9	96.1+/-15.0
50S ribosomal protein L2	1	273.8+/-10.2
50S ribosomal protein L11	1	144.4+/7.0
50S ribosomal protein L23	2	99.2+/-10.3
50S ribosomal protein L29	7	68.2+/-9.8
elongation factor P	1	185.4+/-16.9
flap-1 endonuclease	2	832.6+/-204.1
translation initiation factor IF-1	4	77.3+/-11.1

1. Search for protein and nucleotide against RefSeq genomes (Aug. 10, 2010) identified cases where gene/protein were not present as either normal or non-functional. In those cases, a new gene/CDS/protein was added to the RefSeq record.

2. Protein name/functional name.

3. Number of proteins added for each category, in some cases multiple additions to the same genome.

4. The average protein length and standard deviation of lengths for all proteins from all clusters for each functional group. In some cases there are multiple protein clusters for one functional group.

The core set establishes the initial set for functional name comparison for the 61 functions and 191 clusters. Comparison to TIGRFAM, HAMAP, and KEGG resulted in mapping to 127, 99, and 77 families (or subfamilies), respectively. A total of 122 of the 191 clusters have mappings to all other sources. Of those, only 26 have identical curated names. Multi-way comparison shows that most non-identical names are synonymous, except in a few cases. Examples include the tRNA synthetases, which almost always have identical names, but in a few cases are named as the ligase and not the synthetase. An example is 'tryptophanyl-tRNA synthetase' which in some instances is named 'tryptophan--tRNA ligase' the accepted NC-IUB (Nomenclature Committee of the International Union of Biochemistry) name for the Enzyme Commission number 6.1.1.2 (Table 1). Pairwise comparison of ProtClustDB clusters and the other protein family sources shows two things: 1) a number of protein family resources are missing curated core functions or that these families mapped below threshold levels, and 2) that there are substantially higher numbers of identically curated protein names in two- and three-way comparisons. All four databases have agreed to resolve differences and to work to incorporate the UniProt guidelines into the curated functional names. As these resources are heavily used in genome annotation pipelines, improvements to these records will improve annotations in many genomes and set a standard for other resources. Additional protein family resources are encouraged to be included if they agree to the same goals and are welcome to contact us. InterPro, for example, is another database that integrates information from a variety of source databases and their ongoing effort was acknowledged at the workshop [80].

### Viral/phage annotation standards

Viral annotation standards were discussed for the first time at the 2010 annotation workshop. A set of proposals was published separately and synthesizes many of the ideas presented above with respect to issues of annotation, capturing experimental data, meta-data, and genome classification, all in the context of viral genomes [81].

## Conclusions

These guidelines provide mechanisms for individual researchers studying a single genome as well as those doing high throughput sequencing to ensure that high quality annotation is produced, submitted to, and available from the sequence archives. Mechanisms are in place to capture annotation methodologies and evidence, and in conjunction with standards developed by other international bodies where meta-data submission has been defined, provide a rich and understandable way to determine exactly how annotation was produced. Standard protein naming guidelines and projects to compare and update protein naming resources will result in higher quality annotation resources and protein names in submitted genomes. A major goal of setting minimal standards for the annotation and submission of gold standard complete genomes was achieved and will elevate that set of fundamentally important resources for all researchers, ensuring those studying basic biological processes, epidemiological outbreaks, and large-scale metagenomic projects will have a high quality resource to draw from when making hypotheses and drawing inferences (Table 6). Although not all issues were resolved, and many more remain to be addressed at future workshops, these initial guidelines provide a blueprint for a way forward to resolving these issues and we recognize that many others are working towards similar or parallel goals. One such project is the COMBREX initiative to establish a gold standard set of functionally annotated proteins as well as a source of predictions against which functions can be tested [82]. If complete genomes are to be efficiently utilized as reference genomes it is essential that they represent the highest quality annotation possible. Although this document specifically listed efforts by NCBI to provide resources and tools to improve annotation, NCBI recognizes the ongoing work to improve annotation by all of the organizations that attended and contributed to all workshops.

**Table 6:** Minimal annotation standards and guidelines accepted At 2010 NCBI genome annotation workshop^1^

**1. A complete prokaryotic genome should have:**
a. set of ribosomal RNAs (at least one each 5S, 16S, 23S)
b. a set of tRNAS (at least one each for each amino acid)
c. protein-coding genes at expected density (not all named 'hypothetical protein' and all core genes annotated)
**2. Annotations should follow INSDC submission guidelines:**
Annotation standards should follow feature table format and submission guidelines (GenBank/ENA/DDBJ - Table 1)
a. prior to genome submission a submitted Bioproject record with a registered locus_tag prefix is required and the genome record should contain the Bioproject ID. All proper features should have genes and locus_tags
b. the genome submission should be valid according to feature table documentation and follow the standards
**3. Methodologies and SOPs (Standard Operating Procedures):**
Information about SOPs and additional meta data can be provided in a structured comment with more specific information about experimental or inference support provided on annotated features (see Table 2).
**4. Exceptions:**
Exceptions (unusual annotations, annotations not within expected ranges - see Table 1) should be documented on the genome record and strong supporting evidence should be provided.
**5. Pseudogenes:**
Annotated pseudogenes should follow the accepted formats (see Table 4).
**6. Additional/enriched annotations:**
Additional (enriched) annotations should follow INSDC guidelines, and be documented as above (SOPs and evidence).
**7. Catalog of reputable annotation guidelines, software, and pipelines:**
This non-exhaustive list of reliable software, sources, and databases for the production of microbial genome annotation is a useful community resource that aids in producing high quality genome annotation (Table 1).
**8. Validation checks and annotation measures:**
Validation checks should be done prior to the submission of a new genome record. NCBI has already provided numerous tools to validate and ensure correctness of annotation and additional checks and reports will be put in place to ensure minimal standards are met (see Table 1).

^1^ Guidelines were created for complete genomes (all replicons closed to single contigs). In some cases the minimal set of annotations will not be found on draft genomes, but the guidelines for annotation still apply.
